# Phenolic Acid Functional Quaternized Chitooligosaccharide Derivatives: Preparation, Characterization, Antioxidant, Antibacterial, and Antifungal Activity

**DOI:** 10.3390/md21100535

**Published:** 2023-10-13

**Authors:** Yan Sun, Jingmin Cui, Liguang Tian, Yingqi Mi, Zhanyong Guo

**Affiliations:** 1 Key Laboratory of Coastal Biology and Bioresource Utilization, Yantai Institute of Coastal Zone Research, Chinese Academy of Sciences, Yantai 264003, China; sunyan20@mails.ucas.ac.cn (Y.S.); jmcui@yic.ac.cn (J.C.); yqmi@yic.ac.cn (Y.M.); 2 University of Chinese Academy of Sciences, Beijing 100049, China; 3 Shandong Saline-Alkali Land Modern Agriculture Company, Dongying 257300, China; 4 Yantai Agricultural Technology Extension Center, Yantai 265499, China; tianliguang@yt.shandong.cn

**Keywords:** chitooligosaccharide, chitooligosaccharide quaternary ammonium salt derivatives, phenolic acid, antioxidant activity, antibacterial activity, antifungal activity

## Abstract

As a promising biological material, chitooligosaccharide (COS) has attracted increasing attention because of its unique biological activities. In this study, fourteen novel phenolic acid functional COS derivatives were successfully prepared using two facile methods. The structures of derivatives were characterized by FT-IR and ^1^H NMR spectra. The in vitro antioxidant activity experiment results demonstrated that the derivatives presented stronger 1,1-Diphenyl-2-picryl-hydrazyl (DPPH), superoxide, hydroxyl radical scavenging activity and reducing power, especially the *N,N,N*-trimethylated chitooligosaccharide gallic acid salt (GLTMC), gallic acid esterified *N,N,N*-trimethylated chitooligosaccharide (GL-TMC) and caffeic acid *N,N,N*-trimethylated chitooligosaccharide (CFTMC) derivatives. Furthermore, the antifungal assay was carried out and the results indicated that the salicylic acid esterified *N,N,N*-trimethylated chitooligosaccharide (SY-TMC) had much better inhibitory activity against *Botrytis cinerea* and *Fusarium graminearum*. Additionally, the results of the bacteriostasis experiment showed that the caffeic acid esterified *N,N,N*-trimethylated chitooligosaccharide (CF-TMC) had the potential ability to inhibit *Escherichia coli* and *Staphylococcus aureus* bacteria. Altogether, this study may provide a neoteric method to produce COS derivatives with significantly increased biological activities, which have potential use in food, medicine, and health care products and other related industries.

## 1. Introduction

Chitosan is the only natural cationic basic polysaccharide on earth and the second most abundant natural polysaccharide after cellulose [1]. Chitooligosaccharide (COS) is the degradation product of chitosan and usually produced by acid hydrolysis, physical hydrolysis or enzymatic degradation methods [2]. It is mainly a linear oligomer of glucosamine linked by a β-1,4 glycosidic bond, with a small amount of acetylglucosamine. The degree of polymerization ranges from 2 to 20 [3]. Due to the low degree of polymerization, COS has more excellent biological activities compared with chitosan. Previous research confirmed that COS not only has good water solubility [4], biocompatibility and biodegradability, but also has anti-inflammatory, anticancerogenic, antidiabetic, antimicrobial, anti-HIV-1, antioxidant, antiangiogenic, neuroprotective, and immunostimulatory effects [5,6,7,8,9]. Owning to these good activities, COS is becoming the focus of studies to develop products that can be used for humans, such as in the medicine, food, cosmetic, health care and agriculture industries [10]. Among the various studies, chemical modification is one of the most important research directions as the amino and hydroxyl groups of COS provide possible reaction sites to synthesize novel compounds [11]. And favorable substitution can further improve the biological activity and utilization value of COS.

Phenolic acids are natural products that are derived from plant secondary metabolism [12]. They are widely found in numerous plants and are easy to obtain [13]. Studies have shown that phenolic acids possess excellent antioxidant activity. The hydroxyl group of phenolic acids is the main functional group that exerts antioxidant activity because it can deliver hydrogen atoms to free radicals, interfere with the chain propagation reactions and chelate metal ions. In addition, the structural diversity of phenolic acids can affect the antioxidant capacity. Phenolic acids also have other biological activities, such as anti-inflammatory, immunoregulatory, anti-allergic, anti-atherogenic, anti-microbial, anti-thrombotic, cardioprotective, anti-cancer and antidiabetic properties [14,15,16,17,18]. Based on these multiple biological activities, phenolic acids are becoming a kind of potential biomaterial that can be used in the pharmaceutical, food and biomedicine industries, among others [19]. However, the low water solubility of phenolic acids limits their application.

So, based on the above characteristics of COS and phenolic acids, we want to synthesize new compounds with their combined activities. There has been much research on the synthesis of phenolic acid chitosan or COS derivatives. Tae-kil et al. selected eight phenolic acids (protocatechuic, 4-hydroxybenzoic, vanillic, syringic, *p*-coumaric, caffeic, ferulic and sinapinic acid) to synthesize eight acylated COS derivatives by the DCC/HOBt grafting system and demonstrated that caffeic acid-c-COS had the highest antioxidant activity [20]. Thanh et al. adopted a DCC grafting system to synthesize gallic acid acylated chitooligosaccharide and verified that it has a more enhanced inhibitory effect against allergic reactions in RBL-2H3 mast cells than COS [21]. Chen et al. prepared gallic acid grafted carboxymethyl chitosan through an ascorbic acid/hydrogen peroxide initiated graft copolymerization reaction and proved it can notably modulate intestinal microcirculation [22]. Sun et al. prepared 14 phenolic acid COS derivatives using seven phenolic acids and demonstrated that the derivatives had stronger antioxidant activity than COS [23]. Dai et al. used gallic acid to synthesize gallate-COS via carbodiimide and proved it had obvious antioxidant activity [24]. Mi et al. selected eight organic acids (cumaric acid, ascorbic acid, ferulic acid, p-coumaric acid, caffeic acid, gallic acid, salicylic acid, hydroxybenzoic acid) and synthesized eight hydroxypropyltrimethyl ammonium chitosan derivatives by the ion exchange method. The antioxidant activity test demonstrated these derivatives had dramatic enhancements in free radical scavenging activity [25]. Xing et al. prepared several organic acid COS salts by microwave method and tested their effect on NO secretion of macrophages. The results demonstrated that different salt chitooligosaccharides had different effects on promoting NO secretion [26]. To sum up, phenolic acid grafted chitosan or COS used to prepare new compounds can further enhance their biological activities, and the new compounds have great application potential. Moreover, quaternized chitosan is a widely used chitosan derivative. Because of the increased positive charge density, the quaternized chitosan has much better biocompatibility, solubility, antioxidant and antibacterial activities than chitosan [27]. Therefore, we speculate that the biological activity of derivatives prepared by quaternizing COS and grafting phenolic acids could also be improved.

In this study, we designed two synthetic routes and chose seven phenolic acids to prepare quaternized chitooligosaccharide phenolic acid derivatives. First, the *N,N,N*-trimethylated chitooligosaccharide phenolic acid salt derivatives were synthesized by ion exchange methods: the amino group of COS was first quaternized and then different phenolic acids were introduced through ion exchange. Seven derivatives were synthesized successfully: *N,N,N*-trimethylated chitooligosaccharide gallic acid salt (GLTMC), *N,N,N*-trimethylated chitooligosaccharide ferulic acid salt (FUTMC), *N,N,N*-trimethylated chitooligosaccharide *p*-coumaric acid salt (CMTMC), *N,N,N*-trimethylated chitooligosaccharide caffeic acid salt (CFTMC), *N,N,N*-trimethylated chitooligosaccharide protocatechuic acid salt (PCTMC), *N,N,N*-trimethylated chitooligosaccharide sinapic acid salt (SPTMC), *N,N,N*-trimethylated chitooligosaccharide salicylic acid salt (SYTMC). Second, the phenolic acid esterified *N,N,N*-trimethylated chitooligosaccharides were synthesized by the CDI catalytic system: the carboxyl group of phenolic acids was catalyzed by CDI and then reacted with the hydroxyl group of *N,N,N*-trimethylated chitooligosaccharide to form an ester bond. Seven derivatives were finally synthesized: gallic acid esterified *N,N,N*-trimethylated chitooligosaccharide (GL-TMC), ferulic acid *N,N,N*-trimethylated esterified chitooligosaccharide (FU-TMC), *p*-coumaric acid esterified *N,N,N*-trimethylated chitooligosaccharide (CM-TMC), caffeic acid esterified *N,N,N*-trimethylated chitooligosaccharide (CF-TMC), protocatechuic acid esterified *N,N,N*-trimethylated chitooligosaccharide (PC-TMC), sinapic acid esterified *N,N,N*-trimethylated chitooligosaccharide (SP-TMC) and salicylic acid esterified *N,N,N*-trimethylated chitooligosaccharide (SY-TMC). The structures and thermal stability of these derivatives were characterized by FT-IR, ^1^H NMR spectra and thermal analysis methods. The degree of substitution in derivatives was also calculated and analyzed by ^1^H NMR spectra. Moreover, the antioxidant activity of these derivatives was evaluated by the DPPH radical scavenging activity, superoxide radical scavenging activity, the hydroxyl radical scavenging activity and the reducing power experiments in vitro. Antifungal and antibacterial assay experiments were also implemented to detect the antimicrobial activities of derivatives. In addition, the cytotoxicity of derivatives was tested by L929 cells using the MTT method in vitro. This study may provide a novel method to synthesize COS phenolic acid derivatives, and the new green substances prepared in this paper can serve as antimicrobial or antioxidant agents for use in many fields.

## 2. Results and Discussion

### 2.1. Chemical Synthesis and Characterization

**Scheme 1:**
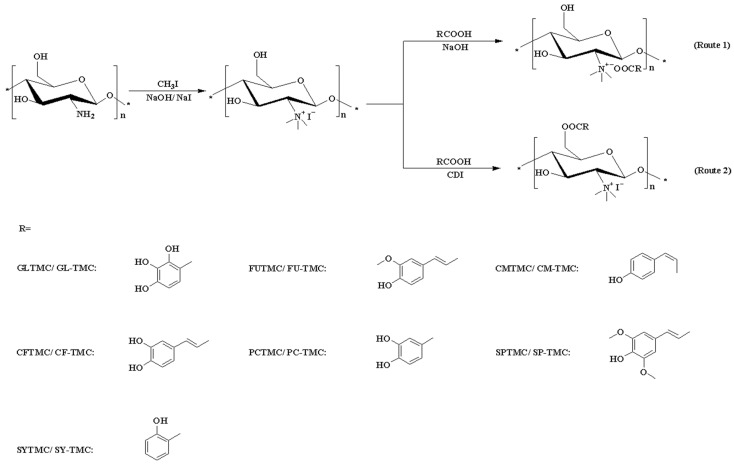
Synthesis routes for phenolic acid COS derivatives.

The synthesis procedure for phenolic acid TMC salt derivatives is shown in Route 1 of Scheme 1. In the first step of the reaction, in the presence of NaI and NaOH, TMCI was formed by the COS and CH_3_I reaction. In the second step of the reaction, phenolic acids reacted with NaOH and formed a carboxylate anion. After TMCI was added, the carboxylic acid anion of phenolic acid replaced I^-^ and combined with the dissociated TMC cation by electrostatic attraction through ion exchange to form the desired products [28].

The synthesis procedure for the phenolic acid esterified TMC derivatives is shown in Route 2 of Scheme 1. CDI is an imidazole derivative with high reactive activity due to the structure of double heterocycles. Herein, CDI reacted with the carboxyl groups in phenolic acids to form carbonyl imidazole. After TMCI was added, carbonyl imidazole reacted with the hydroxyl group of TMCI to form an ester bond; thus, the desired products were obtained [29,30].

### 2.2. FT-IR Spectra

**Figure 1:**
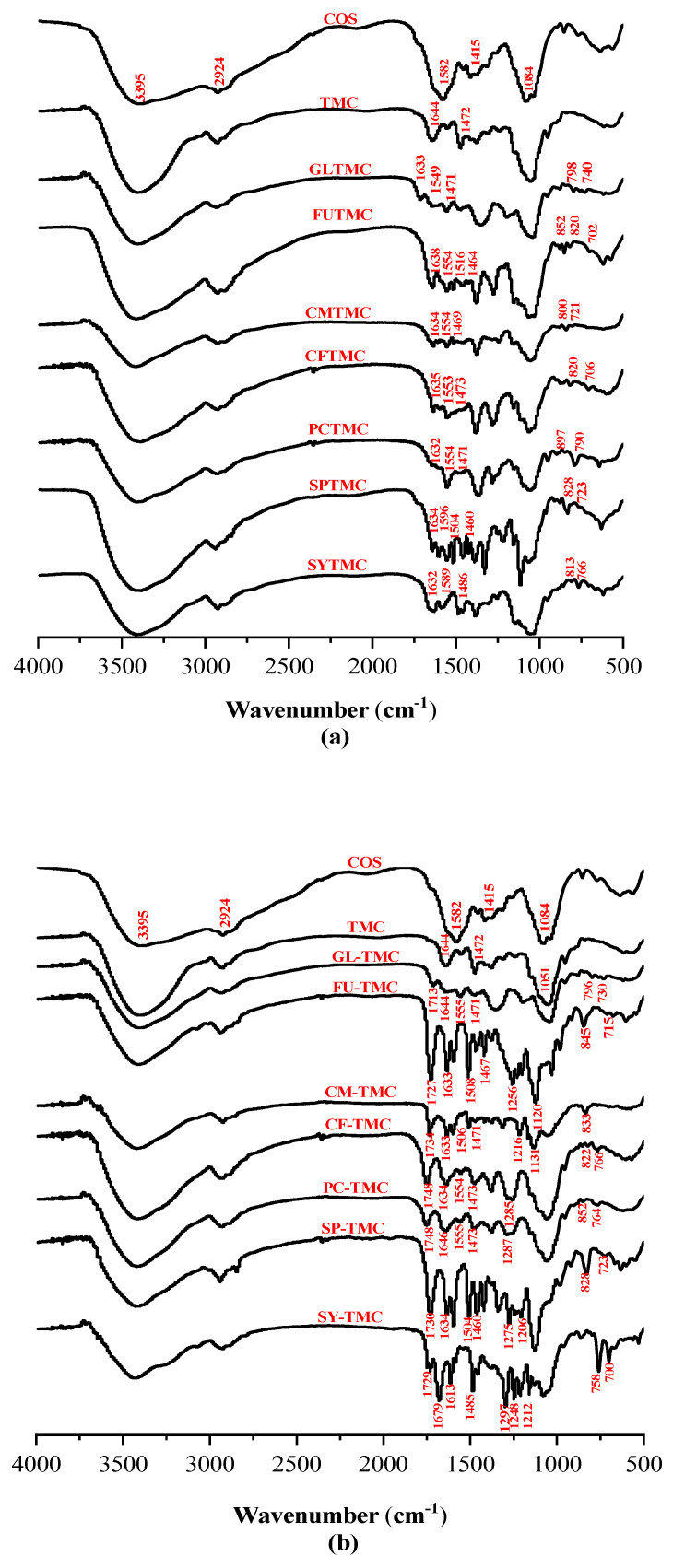
(**a**) FT-IR spectra of COS, TMCI and TMC phenolic acid salt derivatives; (**b**) FT-IR spectra of COS, TMCI and phenolic acid esterified TMC derivatives.

The FT-IR spectra of the TMC phenolic acid salt derivatives are shown in Figure 1a. As exhibited in the figure, for COS, the wavenumber of 3395 cm^−1^ can be attributed to the O-H and N-H bending, and the 2924 cm^−1^ wavenumber is the stretching vibration of O-H. The band at 1582 cm^−1^ represents the amino group and the characteristic peak at 1415 cm^−1^ is the deformation vibration of -CH_2_ and -CH_3_. The absorption peak at 1084 cm^−1^ is the C-O-C stretching vibration [31]. For TMCI, a new spike appearing at 1472 cm^−1^ can be attributed to the -N^+^(CH_3_)_3_, and the absorbance at 1644 cm^−1^ can be assigned to the band of N-CH_3_ [32]. This demonstrates the successful synthesis of the TMCI derivatives. Moreover, for the TMC phenolic acid salt derivatives, the spikes at 1470 cm^−1^ and 1640 cm^−1^ are obvious, which prove the presence of N^+^(CH_3_)_3_. In addition, the new spikes at 1549, 1516, 1554, 1554, 1553, 1554, 1504, 1596 and 1589 cm^−1^ of GLTMC, FUTMC, CMTMC, CFTMC, PCTMC, SPTMC and SYTMC can be attributed to the characteristic absorption of the C-C stretching vibration of the benzene ring [33]. Meanwhile, the peaks appearing at GLTMC (740, 798 cm^−1^), FUTMC (702, 820, 852 cm^−1^), CMTMC (721, 800 cm^−1^), CFTMC (706, 820 cm^−1^), PCTMC (790, 897 cm^−1^), SPTMC (723, 828 cm^−1^) and SYTMC (766, 813 cm^−1^) are the C-H out-of-plane bending vibrations of the benzene ring [34]. Comprehensively, the FT-IR spectra characterization primarily demonstrates that the derivatives were successfully synthesized.

The FT-IR spectra of phenolic acid esterified TMC derivatives are shown in Figure 1b. It can be seen that the derivatives retain the characteristic absorption of COS and TMCI. The reaction displays an obvious characteristic spike around 1720 cm^−1^, which can be attributed to the C=O stretching vibration of the ester bond. And the absorption at 1280–1050 cm^−1^ can be assigned to the C-O-C stretching vibration of the ester bond, which proves the successful synthesis of esterified derivatives. Furthermore, the spikes at 1555 (GL-TMC), 1508 (FU-TMC), 1506 (CM-TMC), 1554 (CF-TMC), 1555 (PC-TMC), 1504 (SP-TMC) and 1613 cm^−1^ (SY-TMC) represent the skeleton vibration absorption peak of the benzene ring, and the spikes at 700–850 cm^−1^ represent the C-H out-of-plane bending vibration absorption peak of the benzene ring. Therefore, the characteristic absorptions demonstrate the successful introduction of phenolic acids.

### 2.3. ^1^H NMR Spectra

**Figure 2:**
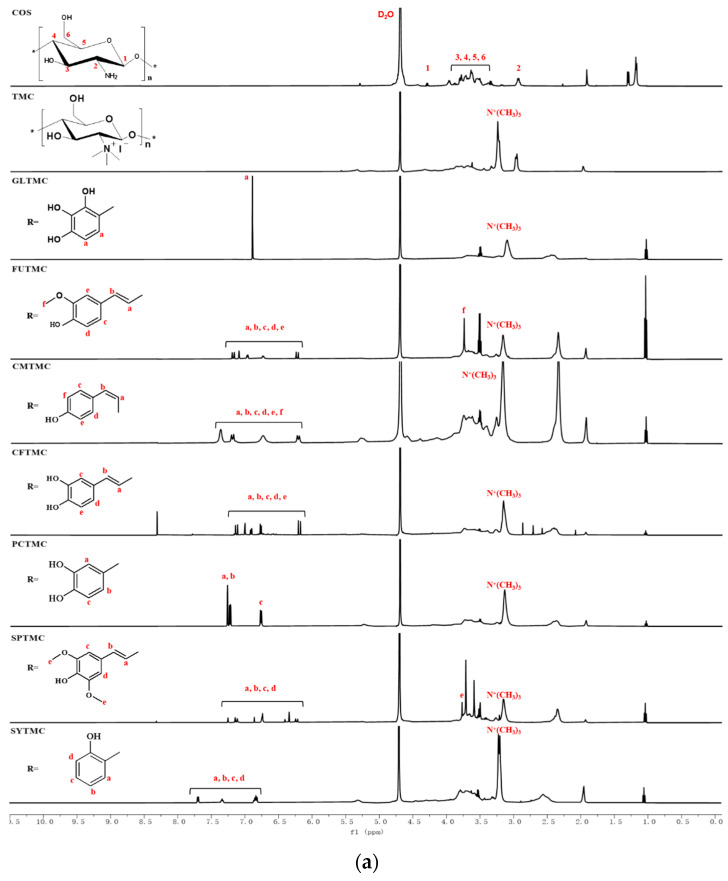

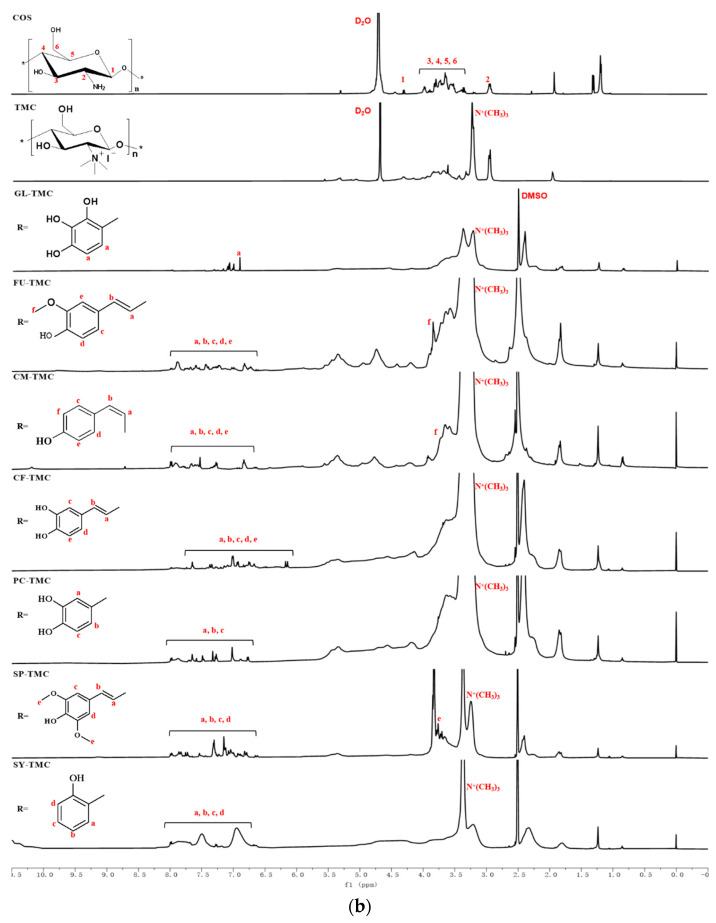
(**a**) ^1^H NMR spectra of COS, TMCI and TMC phenolic acid salt derivatives; (**b**) ^1^H NMR spectra of COS, TMCI and phenolic acid esterified TMC derivatives.

In order to further determine the structures of the products, ^1^H NMR spectra were implemented in this study and the results are shown in Figure 2. The chemical shift at δ 4.79 ppm of the products can be attributed to the D_2_O solvents. The chemical shift at δ 2.50 ppm of the products can be attributed to the DMSO solvents. In addition, the characterization of unmodified COS is as follows: the δ 4.48 ppm can be attributed to [H1], δ 2.69 ppm is [H2] and δ 3.21–4.08 ppm is [H2]-[H6]. As for TMCI, there is an obvious signal at δ 3.22 ppm, which can be assigned to the N^+^(CH_3_)_3_ bond [35]. After the reaction with the phenolic acids, the ^1^H NMR spectra of the derivatives obviously changes. The new shifts appearing at δ 6.0–9.0 ppm prove the successful synthesis of COS phenolic acid derivatives.

### 2.4. Yields and the DS of Chitosan Derivatives

After obtaining the desired products, we calculated their yields, and the result is shown in Table 1. In addition, according to the ^1^H NMR spectra, the [H1] of COS was chosen as the integral standard peak and the DS of each derivative was determined by the ratio of the peak area. The result is also shown in Table 1.

### 2.5. Thermal Gravimetric Analysis (TGA) and Derivative Thermogravimetry (DTG)

**Figure 3:**
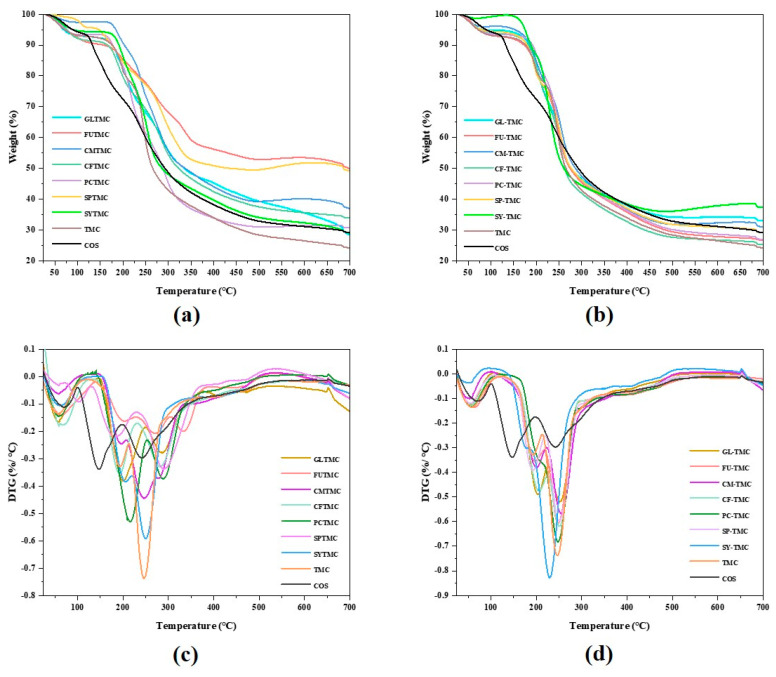
TGA (**a**,**b**) and DTG (**c**,**d**) curves of COS and its derivatives.

With the temperature ranging from 25 to 700 °C, COS had three mass loss steps (Figure 3). The first stage in the range of 30–100 °C with about 8% weight loss was mainly caused by the evaporation of water. And the second stage from 100–200 °C with 17.80% weight loss was caused by the decomposition of the amine and hydroxyl group [36]. The third weight loss in the range of 200–700 °C was mainly caused by the decomposition of the saccharide rings [37]. The final weight loss of COS was about 70.90% of the initial weight. As for the derivatives, it can be seen from the figure that the decomposition temperature of the derivatives was higher compared with COS. In the first stage of 25–200 °C, they obviously showed more stability and less weight loss. In the stage of 200–700 °C, the decomposition rate of derivatives was lower than COS, which demonstrated the better thermal stability. The reason may be that after being introduced into the COS, the phenolic acid formed a hydrogen bond with COS, which strengthened the thermal stability of derivatives. The quaternary ammonium group and the amide group also enhanced the thermal stability of the derivatives. The final mass loss of each derivative was as follows: TMC: 75.80%, GLTMC: 71.74%, FUTMC: 50.1167%, CM-TMC: 63.14%, CFTMC: 66.16%, PCTMC: 69.32%, SPTMC: 50.90%, SYTMC: 71.08%, GL-TMC: 67.02%, FU-TMC: 73.30%, CM-TMC: 69.09%, CF-TMC: 74.81%, PC-TMC: 73.14%, SP-TMC: 70.94%, SY-TMC: 62.66%.

### 2.6. Antioxidant Activity

#### 2.6.1. Scavenging Ability of the DPPH Radical

1,1-Diphenyl-2-picryl-hydrazyl (DPPH), as a nitrogen-centered synthetic radical with a lone pair of electrons, is one of the most common free radicals used to detect the antioxidant activity of substances. As a stable radical, DPPH radical ethanol solution is purple and has a maximum absorption wavelength at 517 nm. When added to the solution, antioxidants can deliver electrons or hydrogen to DPPH to generate the DPPH-H compound, which is more stable, and the color of the solution turns yellow. The degree of discoloration of the solution is quantitatively related to the number of electrons it accepts. The smaller the absorbance is, the stronger the antioxidant capacity of the substance is [38].

**Figure 4:**
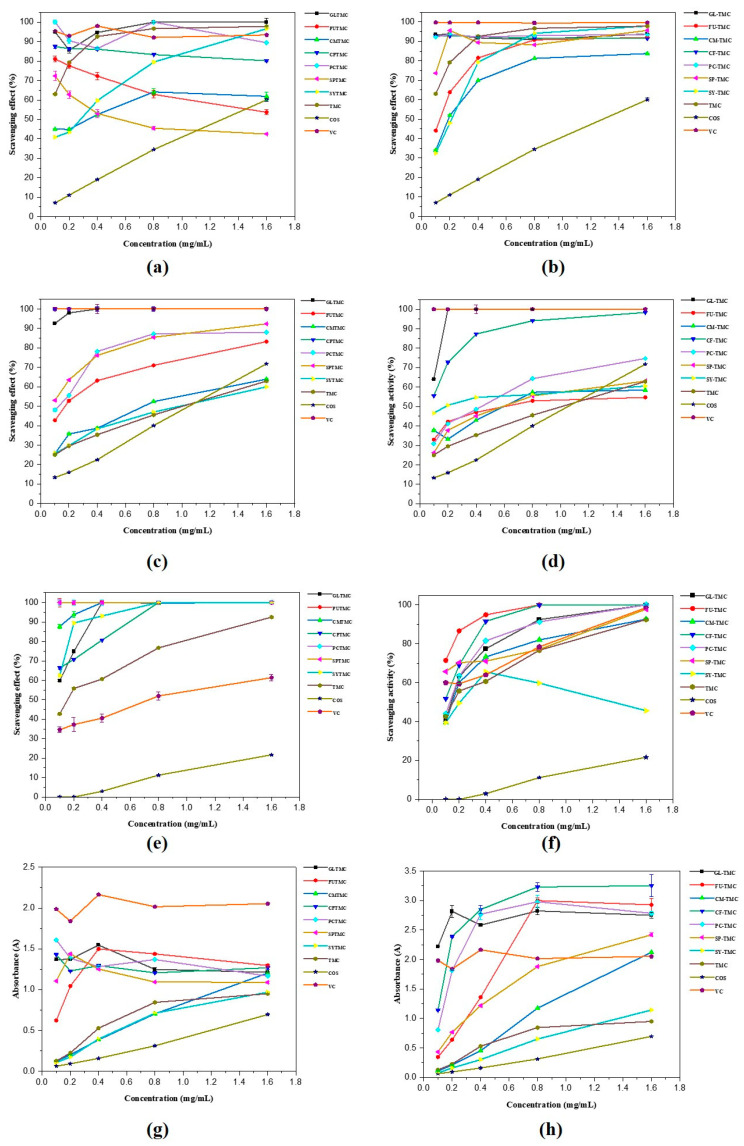
Antioxidant activity of COS and its derivatives: DPPH radical scavenging activity (**a**,**b**); Superoxide radical scavenging activity (**c**,**d**); Hydroxyl radical scavenging activity (**e**,**f**); and reducing power (**g**,**h**).

The DPPH radical scavenging activity of all products is concentration-related (Figure 4a,b). For TMC phenolic acid salt derivatives, it can be observed that the GLTMC, CMTMC, CFTMC, PCTMC, and SYTMC derivatives had obviously enhanced scavenging activity over COS within the range of tested concentrations. And GLTMC had the highest scavenging activity among these derivatives, achieving nearly 100%. So GLTMC is a potentially suitable antioxidant for DPPH radicals. As for FUTMC and SPTMC, at the concentration of 0.10 mg/mL, their scavenging effect reached more than 70%. But with the augmenting of concentration, the scavenging effect declined, and the possible reason may be as follows: with the increase in concentration, the color of the solution gradually deepened, which made its absorbance increase and the scavenging effect decline. As for phenolic acid esterified TMC derivatives, the DPPH radical scavenging activity of all products increased in a dose-dependent manner (Figure 4b). After being modified with phenolic acids, the DPPH radical scavenging activity of COS derivatives was significantly increased. Comprehensively, at the concentration of 1.60 mg/mL, the order of the DPPH radical scavenging activity of derivatives can be ranked as follows: VC > SY-TMC > SP-TMC > GL-TMC > PC-TMC > FU-TMC > CF-TMC > CM-TMC.

#### 2.6.2. Scavenging Ability of Superoxide Radical

A superoxide anion is formed in almost all aerobic cells and is harmful to cellular components as a precursor to more active oxides. Superoxide anion radicals are more dangerous because they have a longer life span and can travel over greater distances than other oxygen radicals [39]. Therefore, it is necessary to develop antioxidants that can effectively scavenge this free radical. In this study, the NADH-PMS-NBT system was used in the superoxide radical scavenging assay experiment. In the presence of NADH and oxygen the in air, PMS can react with them and form a superoxide anion radical. Then the superoxide anion radical reacts with NBT to generate blue substances, which cause the solution to reach the maximum absorption wavenumber at 520 nm. But if there are antioxidants in the system, the superoxide anion radicals will react with the antioxidants first and the solvent will not turn blue, thus the absorbance at 520 nm will decrease. The smaller the absorbance of the solvent, the stronger the superoxide radical scavenging activity [39].

The superoxide radical scavenging ability of the samples is shown in Figure 4c,d. Several conclusions can be drawn from the figure. First, with the increase in concentration, the scavenging activity of all samples was enhanced. Moreover, for the phenolic acid TMC salt derivatives, all derivatives had a higher scavenging effect at the concentrations of 0.10, 0.20, 0.40 and 0.80 mg/mL. GLTMC and CFTMC in particular had outstanding scavenging effects, which reached 100%. The superoxide radical scavenging activity result of phenolic acid esterified TMC derivatives was similar to the salt derivatives. Compared to COS, the antioxidant activity of these derivatives was obviously enhanced. Moreover, among these derivatives, GL-TMC, CF-TMC and PC-TMC had significantly higher antioxidant activity than COS at all tested concentrations and could be developed into excellent antioxidants. The order of the scavenging activity at the concentration of 0.80 mg/mL can be sorted as follows: GLTMC = CFTMC = GL-TMC > CF-TMC > PCTMC > SPTMC > FUTMC > PC-TMC > CM-TMC > SY-TMC > SP-TMC > FU-TMC > CMTMC > SYTMC > TMC > COS. The analysis demonstrates the effective modification of the phenolic acids.

#### 2.6.3. Scavenging Ability of Hydroxyl Radical

The hydroxyl radical is the most reactive free radical, which can react with non-selective compounds such as proteins, nucleic acids, unsaturated fatty acids and almost all biofilms. It can cause damage to macromolecules and organisms [40]. The hydroxyl radical scavenging activity of the samples is shown in Figure 4e,f. As we can see from the figure, augmentation of scavenging activity was identified for all the samples at increasing concentrations. COS had the lowest scavenging effect, which was only 21.66% at the concentration of 1.60 mg/mL. And the scavenging activity of all the derivatives was obviously enhanced at the tested concentrations compared to COS. For example, the scavenging activity of GLTMC reached 100% at the concentration of 0.40 mg/mL.

#### 2.6.4. Reducing Power

Reducing power is usually used to measure the electron-donating capacity of substances, which is also a manifestation of their antioxidant activity [41]. Here the tested system consisted of ferric ions (Fe^3+^). When substances with reducing power are added to the test system, they deliver electrons to Fe^3+^ and are reduced to Fe^2+^. As a result, the color of the solution turns green and has the largest absorbance at a wavelength of 700 nm. The greener the color of the solution, the greater the absorbance value, which represents the stronger reducing power of the substance [42]. So, as the result shows (Figure 4g,h), most of the derivatives had higher absorbance than the COS at tested concentrations, which means a stronger reducing power than COS. On the whole, the reducing power of the derivatives at 1.60 mg/mL concentration can be ranked as follows: CF-TMC > FU-TMC > PC-TMC > GL-TMC > SP-TMC > CM-TMC > VC > FUTMC > CFTMC > GLTMC > CMTMC > PCTMC > SY-TMC > SPTMC > SYTMC > TMC > COS.

In this part, the antioxidant activity of products was measured in vitro by testing the scavenging activity of the DPPH radical, superoxide radical, hydroxyl radical and reducing power. As the above result shows, the antioxidant activity of both the TMC phenolic acid salt derivatives and the phenolic acid esterified TMC derivatives was increased to some extent compared with COS. This demonstrated the significant modification of phenolic acids into COS. Additionally, it can be seen that among these derivatives, GLTMC and GL-TMC had the strongest antioxidant activity, which proves that the number of hydroxyl groups is the key factor that determines the antioxidant activity of phenolic acids. The antioxidant activity of phenolic acids increases with the increase in the hydroxyl group number [43]. Moreover, the structural differences of phenolic acids also affect their antioxidant activity. The existence of the C=C bond in phenolic acids can make the formed free radicals more stable through the resonance effect. And the presence of the methoxy group is also beneficial to improving the antioxidant activity of phenolic acids [44]. So the CFTMC and CF-TMC have much higher antioxidant activity. Furthermore, quaternized COS with positive charges can react with free radicals and is also beneficial to improving the antioxidant activity. Therefore, through this part of experiment, we screened several derivatives with excellent antioxidant activity, which we hope to further develop into new antioxidants.

### 2.7. Antibacterial Activity

With the progress in science and technology and the exploration of human diseases, many antimicrobial drugs have been developed and utilized for humans, which have greatly improved the cure rate of diseases and are beneficial to human health. But because of enhanced bacterial resistance, existing antibacterial agents are facing a serious challenge. Development of novel antibacterial drugs is still a big challenge for humans. *E. coli* is a common Gram-negative bacterium that is widely found in the intestinal tract and other sites in human and animals. Studies have shown that *E. coli* can cause many diseases such as diarrhea, dysentery, urinary tract infection, meningitis and sepsis [45]. *S. aureus* is a typical Gram-positive bacterium that can cause infections and many diseases, seriously threatening human physical health [46]. In this part, we selected these two kinds of pathogenic bacteria and detected the antibacterial activity of COS and its derivatives. The result is shown in Table 2 and Table 3.

Several conclusions can be drawn from the data in Table 2 and Table 3. First, COS and TMCI showed no antibacterial activity. However, after being characterized by phenolic acids, the antibacterial activity of the derivatives was greatly enhanced. Second, the antibacterial activity of different COS phenolic acid derivatives was different. Moreover, the inhibition activity of derivatives against *S. aureus* and *E. coli* was different. For example, the MIC value of GL-TMC against *S. aureus* was 4 mg/mL while against *E. coli* it was 0.125 mg/mL. The possible reason is the difference in the cell wall between *S. aureus* and *E. coli*. Furthermore, among these derivatives, CMTMC and CM-TMC exhibited the best antibacterial activity.

### 2.8. Antifungal Analysis

**Figure 5:**
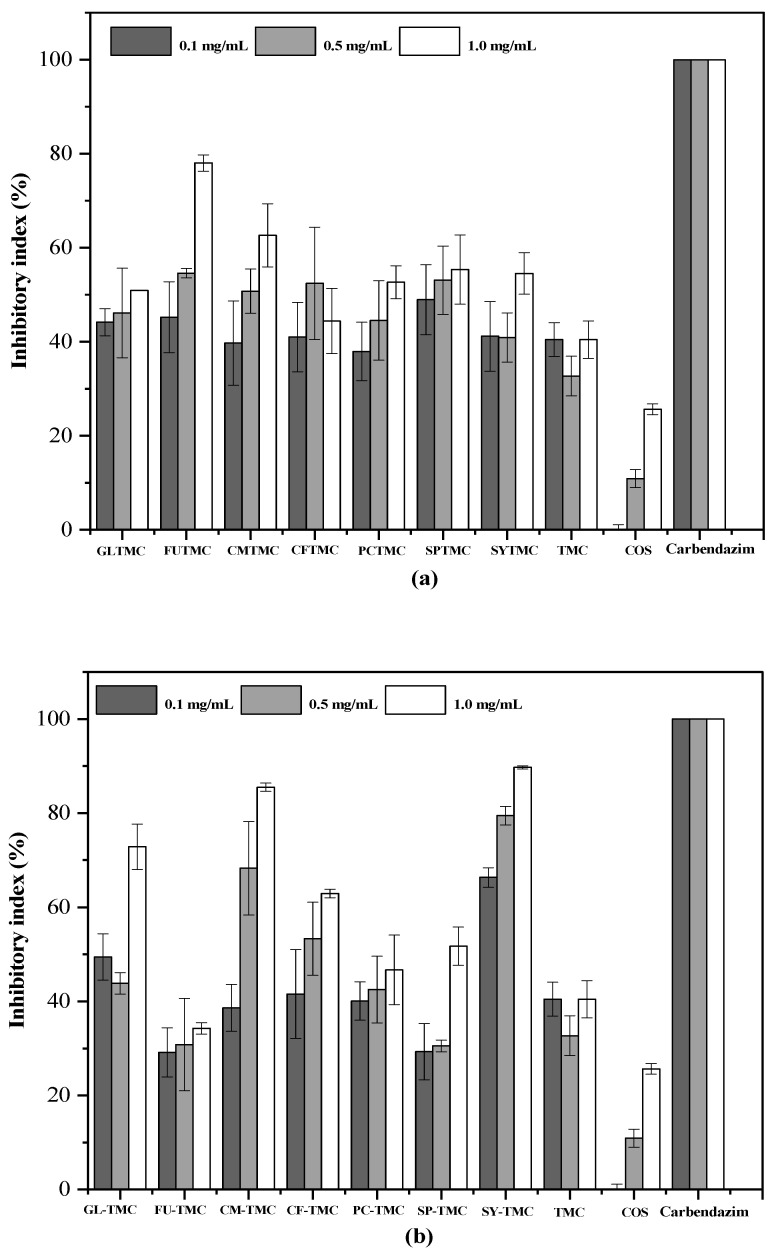

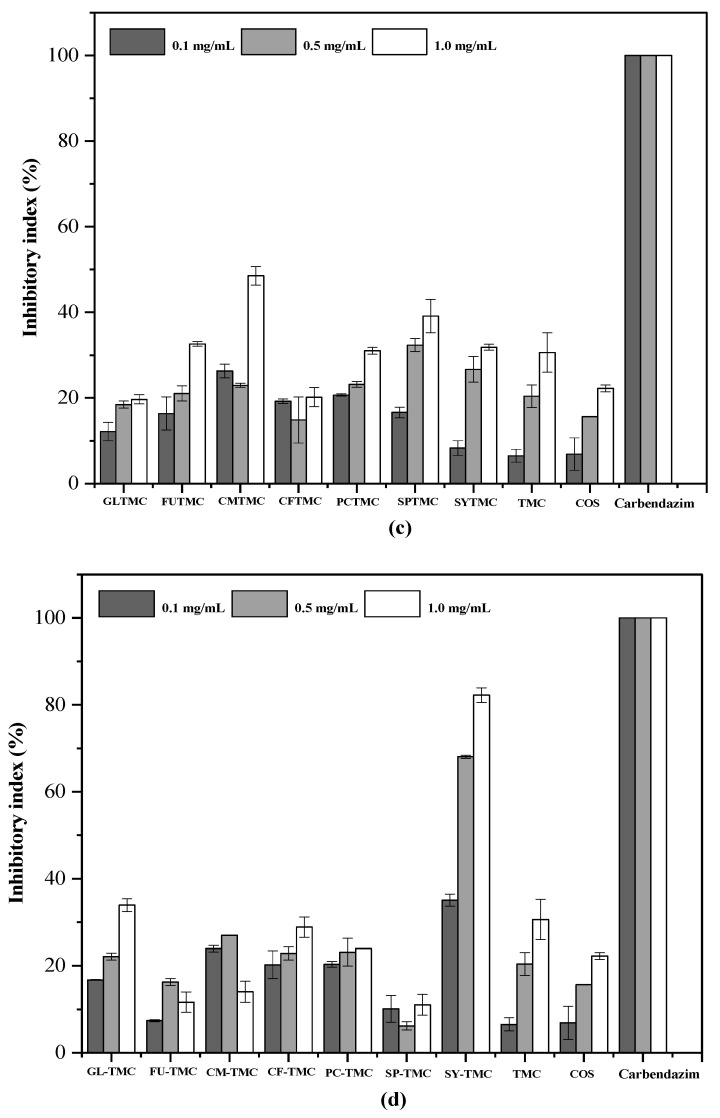
The antifungal activity of carbendazim, COS and its derivatives against *Botrytis cinerea* (**a**,**b**) and *Gibberella saubinetii* (**c**,**d**).

*B. cinerea* is a widely host-borne plant fungus that causes a variety of Botrytis cinerea diseases. It can infest the rhizomes, leaves, flowers and fruits of plants and can be pathogenic at all times of growth. It can cause more than 500 plant pathologies, affecting many economically valuable crops and causing great losses in the postharvest stage of plants. It is one of the most extensively studied necroptotic pathogens [47]. Figure 5a shows the TMC phenolic acid salt derivative inhibitory index of *B. cinerea*. As we can see, carbendazim had the optimal inhibitory effect, where the inhibitory index reached 100%. COS had the lowest inhibitory effect, which was only 25.65% at the concentration of 1.0 mg/mL. As for the derivatives, all the inhibitory indexes were obviously enhanced compared with COS. At the concentration of 0.1 mg/mL, the inhibitory index of the samples can be ranked as follows: SPTMC 48.95%, FUTMC 45.23%, GLTMC 44.17%, SYTMC 41.19%, CFTMC 40.99%, TMC 40.46%, CMTMC 39.73%, PCTMC 37.96%, COS 0.0%. The antifungal activity of the phenolic acid esterified TMC derivatives is shown in Figure 5b. Similar to the TMC phenolic acid salt derivatives, all the derivatives had more enhanced inhibitory activity than COS. Specifically among these derivatives, CM-TMC and SY-TMC presented outstanding inhibitory activity, achieving 85.49% and 89.71%, respectively.

*F. graminearum*, once ranked as one of the top 10 phytopathogenic fungi worldwide, can cause diseases of wheat. Not only does it cause severe wheat yield loss, but its production of mycotoxins can also contaminate cereals, leading to poor grain quality. It causes immune function to decline after human and animal ingestion, causing acute or chronic poisoning, which can seriously harm zoonotic health [48]. The antifungal activity of COS phenolic acid derivatives against *F. graminearum* is shown in Figure 5c,d. As shown in the figure, carbendazim had the best inhibitory activity. The inhibitory index of COS at the concentrations of 0.1, 0.5 and 1.0 mg/mL was 6.86%, 15.24% and 22.86%, respectively. Compared to COS, the derivatives were increased in different degrees. For instance, at the concentration of 1.0 mg/mL, the inhibitory index of each derivative was as follows: GLTMC: 19.67%, FUTMC: 32.62%, CMTMC: 48.54%, CFTMC: 20.19%, PCTMC: 30.04%, SPTMC: 39.13%, SYTMC: 31.85%, GL-TMC: 33.90%, FU-TMC: 11.61%, CM-TMC: 14.01%, CF-TMC: 28.87%, PC-TMC: 23.60%, SP-TMC: 11.03%, SY-TMC: 82.25%, TMC: 30.62%. It can be seen from the data that the antifungal activities of different COS phenolic acid derivatives were different, and the activity of SY-TMC was the most significantly improved. The antifungal activity experiment demonstrated that the introduction of phenolic acids can effectively improve the antifungal activity of COS.

### 2.9. Cytotoxicity Analysis

**Figure 6:**
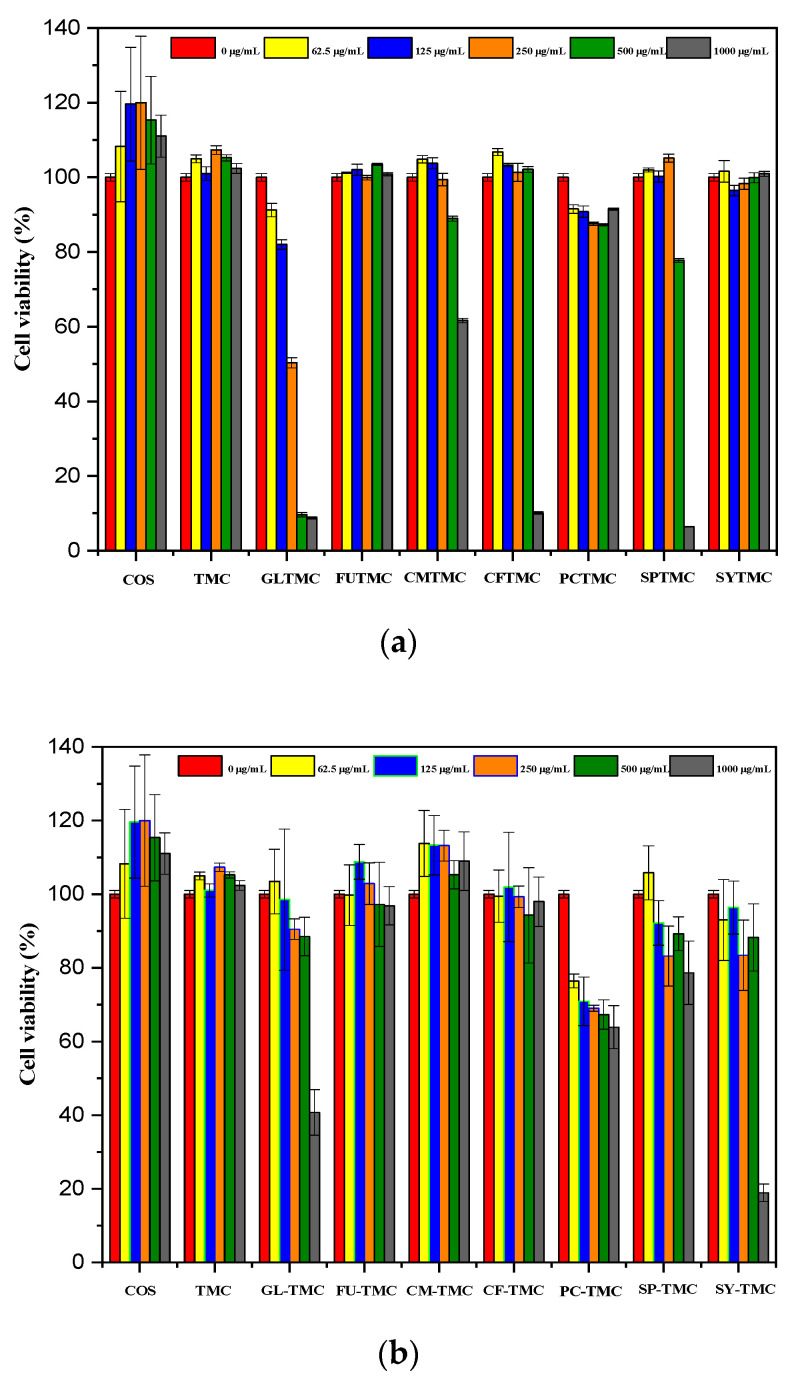
(**a**) the cytotoxicity of COS, TMCI and TMC phenolic acid salt derivatives; (**b**) the cytotoxicity of COS, TMCI and phenolic acid esterified TMC derivatives.

The cytotoxicity of COS phenolic acid derivatives is shown in Figure 6a. As we can see from the figure, when the concentration of the derivatives varied from 62.5, 125, 250, 500 to 1000 μg/mL, the FUTMC, PCTMC and SYTMC derivatives showed a high cell viability, which proves that these derivatives have low cytotoxicity and good biocompatibility. As for other derivatives, CMTMC, CFTMC and SPTMC had high cell viability at concentrations of 62.5, 125, 250 and 500 μg/mL. GLTMC showed an obvious tendency of increasing cytotoxicity with the increase in concentration. The cytotoxicity of phenolic acid esterified TMC derivatives is shown in Figure 6b. It can be observed that FU-TMC, CMTMC, CFTMC and SP-TMC had high cell viability at all tested concentrations. And GL-TMC, SY-TMC had high cell viability at the concentrations of 62.5, 125, 250 and 500 μg/mL, which proves their low cytotoxicity and good biocompatibility. Furthermore, the cell viability of GL-TMC, PC-TMC and SY-TMC showed a descending trend with the increase in concentration. Totally speaking, through this part of the experiment, we screened several derivatives with high antioxidant, antibacterial and antifungal activity and good biocompatibility.

## 3. Materials and Methods

### 3.1. Materials

Chitooligosaccharide (molecular weight of 2000 Da, deacetylation degree of 95%) was purchased from Shandong Weikang Biomedical Technology Co., Ltd. (Linyi, China). L929 cells (GDC0034) were obtained from the China Center for Type Culture Collection. Gallic acid monohydrate, ferulic acid, *p*-coumaric acid, caffeic acid, protocatechuic acid, sinapic acid, salicylic acid, *N,N’*-Carbonyldiimidazole (CDI), sodium hydroxide 1-Methyl-2-pyrrolidinone (NMP) and other used chemical reagents were provided by Sigma-Aldrich Chemical Corp. (Shanghai, China). All chemical reagents were of analytical grade and no further purification was required.

### 3.2. Chemical Synthesis of COS Derivatives

#### 3.2.1. Synthesis of *N,N,N*-Trimethylated Chitooligosaccharide

According to Zhang [49], the synthesis procedure of *N,N,N*-trimethylated chitooligosaccharide (TMCI) was slightly modified and implemented as follows: 20 mmol COS was distributed into 60 mL NMP with 30 mL 15% NaOH solution and 9 g NaI and stirred for 30 min in a round-bottom flask. Then 30 mL CH_3_I was added into the flask drop by drop under freezing conditions. After that, the reaction system was refluxed at 60 °C for 2 h. The TMCI product was precipitated and washed by anhydrous ethanol and anhydrous diethyl ether and obtained after freeze-drying at −50 °C for 24 h.

#### 3.2.2. Synthesis of COS Derivatives—TMC Phenolic Acid Salts

First, 30 mmol NaOH and 30 mmol phenolic acids (gallic acid monohydrate, ferulic acid, *p*-coumaric acid, caffeic acid, protocatechuic acid, sinapic acid, and salicylic acid) were dissolved in 10 mL deionized water and stirred for 2 h. Second, 10 mmol TMCI with 10 mL deionized water was added into the system drop by drop and stirred for 4 h at room temperature. After the reaction, anhydrous ethanol and anhydrous diethyl ether were used to precipitate and wash the products. Finally, the products were obtained after freeze-drying at −50 °C for 24 h [23,25].

#### 3.2.3. Synthesis of COS Derivatives—Phenolic Acid Esterified TMC

First, 20 mmol of different phenolic acids (gallic acid monohydrate, ferulic acid, *p*-coumaric acid, caffeic acid, protocatechuic acid, sinapic acid, and salicylic acid) and CDI was dissolved in DMSO solution and stirred for 12 h at 60 °C. After that, 10 mmol TMCI was added to the reaction system and stirred for 12 h. Finally, the desired products were precipitated and washed with anhydrous ethanol and anhydrous diethyl ether. After freeze-drying at −50 °C for 24 h these derivatives were obtained.

### 3.3. Analytical Methods

#### 3.3.1. Fourier Transform Infrared (FT-IR) Spectroscopy

Fourier transform infrared (FT-IR) spectroscopy (Nicolet IS 50 Fourier Transform Infrared Spectrometer, provided by Thermo Fisher Scientific, Thermo, Waltham, WA, USA) was used at 4000–400 cm^−1^ to analyze the FT-IR structure of the desired products. The products were mixed with KBr disks at a 1:100 ratio and scanned 32 times at 25 °C.

#### 3.3.2. Nuclear Magnetic Resonance (NMR) Spectroscopy

The ^1^H NMR spectra of the desired products were tested and recorded by a Bruke AVIII 500 Spectrometer (500 MHz, Bruker Switzerland, Fällanden, Switzerland) at 25 °C. The products were dissolved in D_2_O or DMSO and the chemical shift values are given in δ (ppm).

#### 3.3.3. Yields and Degree of Substitution (DS) Analysis

In order to estimate the reaction efficiency, the production yield and DS were calculated in this part. Yield represents the ratio (%) of the actual weight (g) of the products to the theoretical weight (g).

The calculation formulation of DS is as follows:

$$DS=H_{s}/H_{1}(\%)$$

where Hs represents the integral area of the hydrogen atom in the benzene ring of the derivatives, H_1_ represents the integral area of the 1-position carbon-bonded hydrogen proton on the main chain of the chitosan.

#### 3.3.4. Thermal Stability

The thermal stability characterization of COS and its derivatives was characterized by a Mettler 5 MP thermogravimetric analyzer (Mettler Toledo, Greifensee, Switzerland). After being vacuum-dried for 24 h, the samples were heated in a range of 30–700 °C at 10 °C /min under a nitrogen atmosphere.

### 3.4. Antioxidant Activity Assay

#### 3.4.1. DPPH Radical Scavenging Activity Assay

According to Li [50], the 1,1-Diphenyl-2-picryl-hydrazyl (DPPH) scavenging activity of products was slightly modified and tested as follows. After lyophilizing to constant weight, the samples were prepared into a solution at an initial concentration of 10 mg/mL. A total of 17.75 mg 1,1-Diphenyl-2-picryl-hydrazyl was dissolved in anhydrous ethanol and placed in a 500 mL volumetric flask. The product solutions were finally diluted into different concentration solutions (0.1, 0.2, 0.4, 0.8, 1.6 mg/mL) with deionized water and 2.0 mL DPPH solution. Then they were incubated together for 20 min and the absorbance of the solution was measured and recorded at 517 nm by a microplate reader. As for the control group, DPPH was replaced by 2.0 mL of the anhydrous group, and the sample was replaced by 1.0 mL deionized water as the blank group. Three replicates for every sample concentration were measured and the scavenging effect was calculated by the following equation:

$$Scavenging\;activity\left(\%\right)=\left[\frac{A_{sample-}A_{control}}{A_{blank}}\right]\times 100$$

where *A_sample_*, *A_control_*, *A_blank_* respectively represent the absorbance of the sample, the control and the blank at 517 nm.

#### 3.4.2. Superoxide Radical Scavenging Activity Assay

The superoxide radical scavenging activity of the products was tested as follows. After lyophilizing to constant weight, the samples to be tested were prepared into a solution at an initial concentration of 10 mg/mL. Tris-HCl buffer (16 mM, pH = 8.0), nicotinamide adenine dinucleotide reduced (NADH, 338 µM NADH dissolved in Tris-HCl buffer), nitro blue tetrazolium (NBT, 72 µM NBT dissolved in Tris-HCl buffer), phenazine methosulfate (PMS, 30 µM PMS dissolved in Tris-HCl buffer) were prepared for the experiment. Measured sample solutions (0.03, 0.06, 0.12, 0.24, 0.48 mL) were added with 0.5 mL NADH, 0.5 mL NBT, and 0.5 mL PMS, and then the volume was fixed to 2 mL with deionized water. After that, the solution was incubated for 5 min under dark condition at room temperature and the absorbance was measured at 560 nm with a microplate reader. In the control group, the NADH was replaced with an equal amount of Tris-HCl buffer, and in the blank group, the sample solution was replaced with an equal amount of deionized water. The scavenging effect was calculated by the following equation:

$$Scavenging\;activity\left(\%\right)=\left[1-\frac{A_{sample}-A_{control}}{A_{control}}\right]\times 100$$

where *A_sample_*, *A_control_* and *A_blank_* respectively represent the absorbance of the sample, the control and the blank at 560 nm [51].

#### 3.4.3. Hydroxyl Radical Scavenging Activity Assay

The hydroxyl radical scavenging activity of products was tested as follows. After lyophilizing to constant weight, the samples were prepared into a solution at an initial concentration of 10 mg/mL. In addition, potassium phosphate buffer (pH = 7.4) H_2_O_2_ (60 µM dissolved in potassium phosphate buffer), EDTA-Fe^2+^ (220 µM, potassium phosphate buffer), and safranine O (0.23 µM, potassium phosphate buffer) were prepared for the experiment. Sample solutions (0.045, 0.09, 0.18, 0.36, 0.72 mL) with 0.50 mL EDTA-Fe^2+^, 1.00 mL potassium phosphate buffer, 1.00 mL safranine O, 1.00 mL H_2_O_2_ solution were incubated without light at 37 °C for 30 min. Meanwhile, the control group contained 1.00 mL deionized water, 0.50 mL EDTA-Fe^2+^, 2.00 mL potassium phosphate buffer, 1.00 mL safranine O, and the blank group constituted 1.00 mL deionized water, 0.50 mL EDTA-Fe^2+^, 1.00 mL potassium phosphate buffer, 1.00 mL safranine O and 1.00 mL H_2_O_2_. Finally, the absorbance of all solutions was measured at 520 nm with a microplate reader, and the scavenging effect was calculated by the following equation:

$$Scavenging\;activity\left(\%\right)=\left[\frac{A_{sample}-A_{blank}}{A_{control}-A_{blank}}\right]\times 100$$

where *A_sample_*, *A_control_* and *A_blank_* respectively represent the absorbance of the sample, the control and the blank at 520 nm [52].

#### 3.4.4. Reducing Power Assay

The reducing power of products was tested as follows. After lyophilizing to constant weight, the samples were prepared into a solution at an initial concentration of 10 mg/mL. In addition, sodium phosphate buffer (pH = 6.6), potassium ferricyanide solution (1%, dissolved in sodium phosphate buffer), trichloroacetic acid (10%, dissolved in sodium phosphate buffer), and ferric chloride solution (0.1%, dissolved in sodium phosphate buffer) were prepared for the experiment. First, sample solutions (0.03, 0.06, 0.12, 0.24, 0.48 mL) and 0.50 mL potassium ferricyanide reacted at 50 °C for 20 min, and then 0.50 mL trichloroacetic acid was added to terminate the reaction. Second, the solution was centrifuged and 0.75 mL supernatant was added into 0.60 mL deionized water with 0.15 mL ferric chloride solution. The reaction system was incubated at room temperature for 10 min. The absorbance of the reaction system was measured at 700 nm and the data represent the reducing power of products [53].

### 3.5. Evaluation of Antibacterial Activity

#### 3.5.1. Preparation of Solutions

The samples were dissolved in sterile water at an initial concentration of 16 mg/mL. Then 100 μL of stock solutions was added to the 96-well plate and continuously diluted 11 times.

#### 3.5.2. Minimum Inhibitory Concentration and Minimum Biocidal Concentration

The antibacterial activity of the samples was tested by the method of minimum inhibitory concentration (MIC) and minimum biocidal concentration (MBC). *Escherichia coli* (*E. coli*) and *Staphylococcus aureus* (*S. aureus*) bacteria were cultivated to test the antibacterial activity, as they were the epitome of Gram-positive bacteria and Gram-negative bacteria, respectively. The experimental process was implemented as follows. First, the bacteria species were incubated in Luria Bertani (LB) medium at 37 °C. And the well-grown bacteria were diluted to 105–106 cell/mL with LB medium. Then the bacterial suspension was added to the 96-well plates containing samples of different concentrations prepared in advance. The volume added to each well was 100 μL, and it was incubated at 37 °C for 24 h. In the meantime, the MIC and MBC were observed by the naked eye. The MIC is the lowest concentration of samples that can completely inhibit visible bacteria growth after 12 h and the MBC is the lowest concentration of the experimental samples that can completely inhibit visible bacteria growth after 24 h. All experimental operations were completed under sterile conditions [54].

### 3.6. Antifungal Assay

Herein, two kinds of plant fungus were cultured to test the antifungal activity of COS and its derivatives. The first one was *Botrytis cinerea* (*B. cinerea*) and the other was *Fusarium graminearum* (*F. graminearum*).

#### 3.6.1. Preparation of Solutions

First, the samples were dissolved in sterile water at an initial concentration of 10 mg/mL. Then the potato dextrose agar (PDA) medium was prepared and sterilized at 120 °C for 25 min to prepare the sample medium. After that, sample solutions of different volumes (2.0, 1.0 and 0.2 mL) were mixed with different volumes of medium (18.0, 19.0 and 19.8 mL) and then poured into disposable Petri dishes. The sample culture medium at concentrations of 0.10, 0.50 and 1.00 mg/mL was formed after solidification. Here in the positive control group, the sample was replaced by carbendazim and the blank control group was deionized water.

#### 3.6.2. Implementation of the Antifungal Experiment

The mycelial growth rate method was used in this experiment. The well-cultivated strains were coated on a PDA medium and cultured in a fungus incubator (28 °C, RH = 60%) for 48 h. After that, a fungi mycelia disk with a diameter of 5 mm was made and carefully inoculated on the sample medium. They were then placed into the fungus incubator for culture. When the mycelia of the blank group grew to the inner edge of the Petri dish, the diameter of the mycelia in all the sample culture media could be measured using the cross crossing method. Three values were measured in parallel for each sample concentration. All experiments were performed under aseptic conditions. Finally, the inhibition rate was measured as follows:

$$Inhibitory\;index\left(\%\right)=\left[1-\frac{D_{s}-5}{D_{b}-5}\right]\times 100$$

where D_s_ is the diameter of the growth zone in the sample plates and D_b_ is the diameter of the growth zone in the blank control plates [37,55].

### 3.7. Cytotoxicity Assay

L929 cells were chosen in this part to test the cytotoxicity of these derivatives by MTT method in vitro. First, L929 cells were incubated in DEMO medium with 10% fetal calf serum and 1% mixture of penicillin and streptomycin at 37 °C with a 5% CO_2_ atmosphere. After passing through twice, the well-incubated cells at a concentration of 5 × 10^4^/mL were added to 96-well plates and incubated for 24 h. Then the derivative solutions at different concentrations were added to the wells and incubated until the cells in control wells multiplied to 90%; the MTT with 0.5 mg/mL was added to the wells and the absorbance was detected at 490 nm with a microplate reader after 4 h with DMSO solution. The cell viability was calculated by the following equation:

$$Cell\;viability=\left[\frac{A_{sample}-A_{blank}}{A_{control}-A_{blank}}\right]\times 100$$

where *A_sample_*, *A_control_* and *A_blank_* respectively represent the absorbance of the sample, the negative control and the blank group at 490 nm.

### 3.8. Statistical Analysis

All experiments above were conducted at least three times and data are presented as the mean ± standard deviation (SD). Significant differences between the groups were assessed using one-way ANOVA followed by Duncan’s test using PASW Statistics 21.0 (SPSS Inc., Chicago, IL, USA). Differences were considered statistically significant at *p* < 0.05.

## 4. Conclusions

In this study, several COS phenolic acid derivatives were successfully synthesized by chemical modification. COS, as a leading compound, was the first to be quaternized and then modified with phenolic acids by two different methods. The structures and DS of these derivatives were characterized by FT-IR and ^1^H NMR spectra. The thermal stability of the derivatives was studied and the results proved they were more stable than COS. Additionally, the antioxidant activity of the derivatives was explored in vitro according to four typical methods. The results demonstrated that the antioxidant activity of the derivatives was greatly improved. Subsequently, the antifungal and antibacterial activities of derivatives were also detected, and among them, derivatives of CMTMC, CM-TMC and SY-TMC had excellent antibacterial and antifungal activities. The cytotoxicity of these derivatives was explored by L929 cells in vitro and the results showed that the cytotoxicity of the derivatives exhibited concentration dependence. Therefore, this study confirms that introducing phenolic acid into COS can significantly improve its biological activity. The derivatives with outstanding activities can be further developed and utilized as antioxidants or bacteriostats in the fields of food, medicine, cosmetics, agriculture and environmental protection. However, the structure–activity relationship and antioxidant mechanism of the products still need further study.

## Figures and Tables

**Table 1:** The yields and DS of COS derivatives.

Compounds	Yields (%)	DS (%)	Compounds	Yields (%)	DS (%)
COS	-	-	TMC	78.57	94.88
GLTMC	86.47	80.00	GL-TMC	58.96	32.00
FUTMC	24.20	66.00	FU-TMC	39.42	43.00
CMTMC	20.40	51.00	CM-TMC	32.40	41.00
CFTMC	68.97	54.00	CF-TMC	59.88	15.00
PCTMC	44.49	36.00	PC-TMC	53.16	12.00
SPTMC	65.42	46.00	SP-TMC	64.34	47.00
SYTMC	73.60	41.00	SY-TMC	73.40	43.00

**Table 2:** The antibacterial activity of COS, TMCI and TMC phenolic acid salt derivatives.

Bacterial Species	Samples									
		COS	TMC	GLTMC	FUTMC	CMTMC	CFTMC	PCTMC	SPTMC	SYTMC
***S. aureus***	**MIC (mg/mL)**	>8	>8	0.5	0.25	0.5	0.25	0.25	0.25	2
	**MBC (mg/mL)**	>8	>8	1	0.5	1	0.5	0.5	0.5	4
***E. coli***	**MIC (mg/mL)**	>8	>8	0.25	1	0.0625	0.5	1	0.5	>8
	**MBC (mg/mL)**	>8	>8	0.5	2	0.125	1	2	1	>8

**Table 3:** The antibacterial activity of COS, TMCI and phenolic acid esterified TMC derivatives.

Bacterial Species	Samples									
		COS	TMC	GL-TMC	FU-TMC	CM-TMC	CF-TMC	PC-TMC	SP-TMC	SY-TMC
***S. aureus***	**MIC (mg/mL)**	>8	>8	4	0.25	0.125	0.25	2	1	0.25
	**MBC (mg/mL)**	>8	>8	8	0.5	0.25	0.5	4	2	0.5
***E. coli***	**MIC (mg/mL)**	>8	>8	0.125	0.125	0.03125	0.5	1	0.5	0.25
	**MBC (mg/mL)**	>8	>8	0.25	0.25	0.0625	1	2	1	0.5

## Data Availability

All data contained in the manuscript are available from the authors.
